# Minimal residual disease in breast cancer: an overview of circulating and disseminated tumour cells

**DOI:** 10.1007/s10585-016-9796-8

**Published:** 2016-05-17

**Authors:** A. Tachtsidis, L. M. McInnes, N. Jacobsen, E. W. Thompson, C. M. Saunders

**Affiliations:** St. Vincent’s Institute, Melbourne, VIC Australia; University of Melbourne, Department of Surgery, St. Vincent’s Hospital, Melbourne, VIC Australia; School of Surgery, The University of Western Australia, Perth, WA Australia; Institute of Health and Biomedical Innovation and School of Biomedical Sciences, Queensland University of Technology, Brisbane, QLD Australia; Translational Research Institute, Woolloongabba, QLD Australia

**Keywords:** Minimal residual disease, Circulating tumour cell, Disseminated tumour cell, Cancer, Metastasis, Epithelial–mesenchymal plasticity

## Abstract

Within the field of cancer research, focus on the study of minimal residual disease (MRD) in the context of carcinoma has grown exponentially over the past several years. MRD encompasses circulating tumour cells (CTCs)—cancer cells on the move via the circulatory or lymphatic system, disseminated tumour cells (DTCs)—cancer cells which have escaped into a distant site (most studies have focused on bone marrow), and resistant cancer cells surviving therapy—be they local or distant, all of which may ultimately give rise to local relapse or overt metastasis. Initial studies simply recorded the presence and number of CTCs and DTCs; however recent advances are allowing assessment of the relationship between their persistence, patient prognosis and the biological properties of MRD, leading to a better understanding of the metastatic process. Technological developments for the isolation and analysis of circulating and disseminated tumour cells continue to emerge, creating new opportunities to monitor disease progression and perhaps alter disease outcome. This review outlines our knowledge to date on both measurement and categorisation of MRD in the form of CTCs and DTCs with respect to how this relates to cancer outcomes, and the hurdles and future of research into both CTCs and DTCs.

## Introduction

Breast cancer is the most commonly diagnosed female cancer with the exclusion of skin cancers. It is the second highest cause of cancer mortality in women behind lung cancer, and carries a lifetime incidence risk of 1 in 8 [1]. Importantly, the proportion of females having survived cancer is greatest for breast cancer, accounting for nearly half of all cancer survivors [2]. This presents an issue with respect to potential relapse and in turn highlights the importance of MRD. Currently, metastatic cancer is incurable and thus metastasis accounts for the majority of deaths associated with cancer. It is well established that the metastatic process is very complex and numerous hypothetical models have been proposed to explain the oft-termed ‘metastatic cascade’ of events, which documents the various requirements of malignant cells escaping the primary tumour and establishing metastases elsewhere [3, 4]. Despite the expanse of knowledge pertaining to the cascade, many exact details are not fully elucidated. It is known that metastases occur preferentially in specific distant sites depending on the organ of origin, such as bone and selected visceral organs for breast cancer [5]. Recurrence can also occur at the primary tumour site even after complete tumour resection with apparently clear surgical margins. Recurrence or metastasis may occur after many years, for example women with hormone receptor positive breast cancer, although having a relatively favourable overall prognosis, still exhibit an elevated annual hazard of recurrence over many years [6]. Comparatively, ‘triple negative’ breast cancers lacking hormone receptors (HRs) and human epidermal growth factor receptor 2 (*HER2*) typically have less treatment options and a higher recurrence rate and thus poorer outcome [7]. However, the majority of recurrences occur in the first 5 years after diagnosis; so long term survivors are more likely to be ‘cured’. This should be considered alongside the fact that old autopsy studies have demonstrated the presence of cancers in people who had otherwise been undiagnosed [8], and that occult metastases undetectable by conventional imaging have been demonstrated in breast cancer patients [9]. This, alongside understanding the metastatic process, is the fundamental basis for the need to study MRD.

Currently an area of great interest is the characterisation of cells that are able to escape from the primary tumour and survive in the peripheral blood as CTCs, or in the bone marrow as DTCs, as is the mechanisms by which they are able to achieve these feats. It has been demonstrated in a model system that around a million cells are shed per gram of primary breast tumour tissue every day but almost all of these are very efficiently eliminated from the circulation within minutes [10]. However, in animal models it has been shown that approximately 2.5 % of cells that are shed are able to survive as micrometastases and approximately 0.01 % can progress to form macrometastases [11, 12]. The question must therefore be asked; what factors give this very small minority of cells the ability to survive and prosper?

This review examines mechanisms of metastasis and the respective role(s) of CTCs and DTCs using breast cancer as a specific example, which in itself is not one disease as is reflected by sub-classification based on both histopathological and molecular characteristics that have been reviewed in depth already [13, 14]. However, there are mechanisms shared with other types of carcinoma and are occasionally discussed here in the context of CTCs/DTCs. We include features of both CTCs/DTCs and aspects of the metastatic sites that enable the dissemination, survival and proliferation of the very small subpopulation of cancer cells that are ultimately able to produce metastases.

## Understanding the metastatic cascade

It is known that cancer cells are typically heterogeneous. This has been demonstrated in vitro and in vivo both molecularly [15–18] and proteomically [19–21], and underpins the associated phenotypic heterogeneity of cancers. For example, even within the same breast cancer and in cases of metastases, there appears to be a subset of putative ‘cancer stem cells’ (CSCs) that are intrinsically highly resistant to chemotherapy and/or radiotherapy and that are involved in the crucial step of cancer dissemination [22–24]. This heterogeneity has been attributed to the widely accepted clonal evolution theory in cancer, which describes a sequential accumulation of favourable mutations over time that may either be key drivers or potentially beneficial passengers in cancer development. Aside from the CSC theory discussed later, recent work has presented a new idea in the context of colorectal cancer that may have application in other carcinomas. Sottoriva et al. [25] have proposed the big bang model that explains intra-tumoural heterogeneity (ITH) as being a consequence of both broad clonal and more isolated sub-clonal mutations within a tumour during early development. The emphasis on these sub-clonal mutations occurring early is of importance with respect to ITH, as it is suggested that they are more likely to mix and then spatially spread as the tumour grows. Thus, timing is considered of importance in this model as the prevalence of these sub-clonal mutations in a given tumour is proposed to occur not because they provided a selective advantage, but as a consequence of when they arose. In the context of colorectal cancer this idea of many sub-clonal mutations occurring in parallel early on makes sense given the abundance and highly active nature of proliferating cells within the crypts of Lieberkuhn, however other tissues do not share this similar architecture/behaviour and therefore the applicability of the big bang model in other cancers remains to be seen. It is generally accepted and understood that there is a progression of events that must occur for a primary tumour in the breast to become established at a distant metastatic site. Initially there is an activation of signalling pathways that control tumour cytoskeletal dynamics, the turnover of cell–matrix and cell–cell junctions and subsequently, active tumour cell migration into the surrounding tissue [26]. Malignant cells must then intravasate into blood or lymphatic vessels, penetrate basement membranes and endothelial walls, survive whilst in the circulation, evade immune defences and other cell death mechanisms such as apoptosis, and travel to a secondary site [27–29]. At this point they must extravasate into the distant tissue and regain or enhance cellular characteristics that allow for anchorage, communication, survival and adaptation into the new microenvironment, in turn promoting mechanisms that enable the proliferation of a cohesive mass of tumour cells which will ultimately become an overt macrometastasis [29].

## Seed and soil hypothesis

CTCs are ‘in transit’ cancer cells arising initially from the primary tumour, but later from micrometastases (when there is no clinical evidence of metastasis) and from overt metastases. CTCs, which have been predominantly studied and observed in the vascular bloodstream rather than lymphatics, were originally discovered in 1869 by Australian physician Thomas Ashworth [30]. Shortly thereafter, in 1889, Stephen Paget observed that the process of metastasis did not seem to occur by chance and proposed the ‘seed and soil’ hypothesis [31]. The ‘seed’, or CTC as we currently know it to be, would be able to grow and establish a new tumour only if able to locate the appropriate ‘soil’ in which to propagate. Paget based this hypothesis on the post-mortem examination of 735 breast cancer patients, where he noted that there was a distinct preference for metastatic sites such as bone and selected visceral organs [31].

This hypothesis has subsequently been repeatedly demonstrated with ‘seed’ cells arising from specific tumour types showing a strong preference for the ‘soil’ of specific metastatic sites [32, 33]. It is thought that tumour cells can express particular proteins such as parathyroid-hormone-related peptide (*PTHrP*) [34], or chemokine receptors such as CXC chemokine receptor type 7 (*CXCR7*) [35] and CXC chemokine receptor type 4 (*CXCR4*) [36], which help direct cancer cells toward specific sites like the bone. Additionally, the survival and propagation of tumour cells at a specific secondary site may be determined by chemokines produced at the site of dissemination [22]. Husemann et al. [37] demonstrated early dissemination of cancer cells into the circulation and bone marrow in the context of patients with ductal carcinoma in situ (DCIS) and a model of atypical ductal hyperplasia (ADH), the latter being unexpected as dissemination from ADH has not been seen in patients. They proposed that surgical removal of the primary lesion at very early time points may deprive such early-disseminated cancer cells from systemically-acting factors important for outgrowth and consequently account for dormancy of such cells. They also suggest that primary tumours may secrete factors that prepare the pre-metastatic niche (or ‘soil’) and foster early cancerous colonies. It has been demonstrated by Kaplan et al. that tumour-specific pre-metastatic sites contain bone marrow-derived haematopoietic progenitor cells that express vascular endothelial growth factor (VEGF) receptor 1 (*VEGFR1*) [38, 39]. Work by Kallergi et al. [40] revealed that CTCs in most of the metastatic patients they assessed exhibited an upregulation of *VEGF*. As noted earlier, the same group subsequently found that a *TWIST* mediated epithelial–mesenchymal transition (EMT) also drives an upregulation of *VEGF* [41]. Therefore the presence of *VEGFR1* at the pre-metastatic site may be a crucial factor in the homing of CTCs to bone marrow and the eventual establishment of DTC deposits. This is supported by Kaplan et al. [38], who demonstrated that antibody-mediated inhibition of *VEGFR1* function, or the removal of *VEGFR1* cells from the bone marrow, abrogated the formation of pre-metastatic clusters and prevented tumour metastasis to bone. It was also shown that VEGFR^+^ cells express integrin α_4_β_1_ and that fibronectin is up-regulated in resident fibroblasts by tumour-specific growth factors. Fibronectin is a ligand of integrin α_4_β_1_ and increased expression provides a permissive niche for incoming tumour cells [38]. Interestingly, it has subsequently been shown that α_4_β_1_ osteoclast progenitors respond to VCAM-1 expression by micrometastases, enabling disease progression in bone [42]. Therefore it may be that one critical cell-type is responsible for both metastatic homing and expansion in bone. Similar studies by Gao et al. [43] support a role for bone marrow-derived macrophages conditioning the metastatic niche through the secretion of the proteoglycan versican, which in turn sequesters *TGFβ* and causes reversion from a mesenchymal phenotype (mesenchymal–epithelial transition; MET) in CTCs as they become DTCs.

It is now well established that CTCs can arise from the primary tumour, carry the malignant features of said primary tumour [44], are able to survive in the circulation, have the ability to extravasate and that at least in some patients, a small proportion of them are ultimately able to establish metastases at a distant site, whereby the site itself has been subjected to metastasis-optimising conditions by native cell populations prior to the arrival of the disseminating cancer cell.

## Local mechanisms of disease relapse

### Tumour self-seeding

In addition to establishing metastatic tumours at secondary sites, it has been demonstrated experimentally that CTCs also have the ability to return to the site of tumour origin. Kim et al. [45] were the first researchers to demonstrate this and determined that CTCs (from fluorescently tagged populations) were able to colonise an untagged recipient mammary fat pad (MFP). The source of the CTCs in some instances were from an opposing MFP that had a fluorescently labelled primary tumour growing (of the same cell line), or from fluorescently tagged cells injected directly into the circulation. The study describes an increased capacity for metastatic progeny to be able to re-seed the primary tumour, which coincides with observations by Braun et al. [46] that patients with detectable DTC are at significantly greater risk of local relapse. Interestingly, fluorescently tagged cells from the ‘parental tumour’ that had successfully re-seeded the ‘recipient’ tumour were isolated and determined to have a greater capacity for self-seeding. Furthermore, the transcriptional profile of these ‘seeder’ cells shared similar expression patterns as some of their metastatic counterparts. Mechanisms investigated included the chemo-attractive ability of interleukin 6 (*IL*-*6*) and interleukin 8 (*IL*-*8*), as well as the function of fascin actin-bundling protein 1 (*FSCN1*), matrix metalloproteinase-1 (*MMP1*) and CXC chemokine ligand 1 (*CXCL1*) using the MDA-MB-231 metastatic breast cancer (MBC) cell line model. Therefore ‘self-seeding’ essentially involves attraction of CTCs back to the primary tumour in situ and reflects an ability of CTCs to extravasate and infiltrate the established tumour. Since this publication by Kim et al. [45], other work has been performed attempting to further elucidate the mechanism of self-seeding [47] as well as illustrating its occurrence in osteosarcoma [48]. Moreover, a string of review articles covering the topic of tumour self-seeding also surfaced following the work presented by Kim et al. These review articles, in conjunction with the original work itself, suggest several advantages for tumour self-seeding; (i) an increase in primary tumour growth rate, (ii) the promotion of local re-growth and (iii) the ‘natural selection’ of more aggressive CTC subpopulations that would have greater success at colonising a distant site [22, 49–53]. Therefore this tumour self-seeding process may in fact contribute to the characteristics of CTCs/DTCs, that are still being elucidated and could also be responsible in part for generating the subpopulations of MRD that are more likely to metastasise successfully. Furthermore, work pertaining to tumour self-seeding has yielded data which could at least partly explain observed associations with metastasis, such as; large tumour size, anaplasia (the loss of differentiation and orientation of cells to one another and the surrounding tissue framework), and the hypervascularity of cancers with poor prognosis [45].

### Therapy resistant cells

Work originally investigating putative CSC populations and their behaviour, as will be discussed separately, set the groundwork towards investigations of subpopulations of cancer cell that persist after therapy. A study in 2006 assessed MCF7 and MDA-MB-231 breast cancer cell lines grown in 2D culture compared to CD44^+ve^/CD24^−ve^ subpopulations grown in mammosphere assays after exposure to different amounts of radiation. Greater radioresistance was observed in the subpopulation cultures as measured by reactive oxidative species (ROS) and pH2AX. Further assessment using MCF7 cells revealed that following radiation exposure in 2D culture, non-adherent floating cells were enriched for CD44^+ve^/CD24^−ve^ while adherent cells were not, and that these resilient cells had enhanced Notch1 expression. This resilience was confirmed in 2008 by Fillmore et al. [54] who used a chemotherapeutic approach on a larger number of breast cancer cell lines, but who also performed xenotransplantation assays comparing unsorted and CD44^+ve^/CD24^−ve^/ESA^+ve^ sorted cells and observed a much greater tumour initiating capacity in the sorted subpopulation. In the same year, Li et al. [55] was the first group to assess the CD44^+ve^/CD24^−ve^ phenotype in paired core biopsies from breast cancer patients undergoing neoadjuvant therapy, as well as mammosphere formation capacity. In patients receiving conventional therapy, the proportion of this subpopulation increased in addition to the ability to form mammospheres. However, there was no significant difference in patients receiving lapatinib, and in fact there was a slight reduction. This work was followed by Creighton et al. [56] who used a similar paired biopsy approach in patients that received letrozole or docetaxel neoadjuvant therapy and also observed an increase in the CD44^+ve^/CD24^−ve^ subpopulation as well as mammosphere forming efficiency. Importantly, they demonstrated via immunofluorescence using clinical samples that this resilient subpopulation consisted of hybrid epithelial–mesenchymal cells. Further, that those resilient cells were enriched for mesenchymal markers including *FN1*, *MMP2*, *MMP3*, *FOXC2* (forkhead box protein C2), *VIM*, and *SNAI2*. Since this work there have been publications demonstrating that; (i) these radioresistant cancer initiating cells can maintain self-renewal capacity and are in fact pushed out of quiescence into an actively dividing state [57], (ii) that STAT1 inhibition increases apoptosis post-radiation treatment in CD44^+ve^/CD24^−ve^ MCF7 sorted cells grown in 3D culture [58], and (iii) downregulation of CD44 in this cancer initiating subpopulation increases susceptibility to doxorubicin therapy [59]. Therefore there is evidence to indicate that therapy resilient cells that remain as residual disease are paradoxically being enhanced in their tumour initiating capacity, and in turn a risk with respect to both local tumour recurrence as well as distant metastasis.

## Mechanisms of therapy resistance

Cancer cell resistance of chemotherapeutic agents and radiation therapy have been described as falling into two camps—multi-drug resistance and pan-resistance. While there is overlap with respect to the mechanisms that form the foundation of each process, they are considered as being two distinctly different arms of therapy resistance. The distinction between the two becomes apparent when one considers the way in which resilience is conferred unto the cell, and importantly, the nature of the resistance that results.

### Multi-drug resistance

This is the concept most commonly thought of with regards to therapy resistant cancer cells and revolves around five key mechanisms; (i) drug transporters/efflux pumps, (ii) modulation of apoptosis and senescence pathways by the cancer cell, (iii) cell cycle effects, (iv) mechanical/stochastic factors, and (v) CSC mediated—the latter is discussed in a separate section with respect to EMT and CTCs/DTCs. The ATP-binding cassette (ABC) membrane transporter protein family has for some time been identified as responsible for moving drug out of cancer cells. The major player P-glycoprotein, encoded by the *ABCB1*/*MDR1* gene, has been shown to act on substrates such as anthracyclines, taxanes, vinca alkaloids, and epipodophyllotoxins [60]. However there are substrates that P-glycoprotein acts poorly on, particularly large hydrophilic drugs and nucleoside analogues. Other ABC family proteins aid in acting on different substrates, such as *ABCG2* which is also known as breast cancer resistance protein (BCRP), and acts on amphipathic drugs [61]. Recent work by Jang et al. has demonstrated doxorubicin sensitisation of resilient putative CSC subpopulations from MDA-MB-231, MCF7, and MCF10A cell lines in vitro via downregulation of *ABCG2* as mediated by suppression of adenine nucleotide translocator-2 (*ANT2*) with shRNA. Alteration of senescence pathways makes logical sense in terms of maximising cancer cell longevity, for example by hijacking the telomerase function of the cell as a means of avoiding cellular aging [62]. Theoretically, manipulation of apoptosis pathways would also be of benefit given the fact that therapy often acts to induce cell death due to excessive cell damage, as indeed has been demonstrated in blood cancers [63, 64]. Given that the vast majority of human cancers are carcinomas, which are often devoid of a functional p53 pathway [65], then the role of apoptosis pathway modulation becomes questionable and has indeed been demonstrated to lack functional significance in breast cancer [66]. Conceptually, cell cycle affects are straight forward with respect to cancer cell resilience. Many chemotherapeutic agents and radiotherapy act to disrupt the cell cycle, for example by induction of DNA damage or disruption of microtubule formation needed to complete mitosis, and as such these approaches inherently work best on actively dividing cells. Therefore, cells in a state of quiescence that are not actively dividing would remain unaffected [67]. The final aspect of drug resistance relates to physical parameters that influence drug-target interaction and thus resilience to treatment. If the drug cannot physically interact with its target, then there can be no effect. This has been shown in breast cancer patients who relapsed with brain metastases that were being treated with an antibody that does not cross the blood–brain barrier [68], or in pancreatic ductal adenocarcinoma where drug penetration is poor due to an extensive stromal envelope [69]. The key overall feature of multi-drug resistance is that tumours develop in a way that allows persistence in the face of many, but not all therapies.

### Pan-resistance

Pan-resistance has been described by Borst [70] as being residual cancer disease that persists after initial therapy, which returns in a far more aggressive manner that is completely unresponsive to any treatment. The driving force for pan-resistance is less well defined compared to multi-drug resistance, however the mechanisms already described pertaining to cell cycle effects and CSCs appear to play a potential role. Borst [70] provides two other explanations for pan-resistance, the first being superdefence—whereby the cells work overtime to keep all drugs away from their target. The second is compensation—which does not affect drug-target interaction but rather uses other mechanisms to compensate as a consequence of said interaction, such as activation of parallel pathways to those targeted. Another explanation for the phenomenon of pan-resistance lies in the work of Sharma et al. [71], who used an in vitro model of non small cell lung cancer (NSCLC) treated with an EGFR-targeting tyrosine kinase inhibitor to generate drug-tolerant persisters (DTPs) with 100-fold reduced drug sensitivity. When the DTP cells were grown in the absence of drug they would regain sensitivity, and if left in the presence of drug most would remain quiescent but approximately 20 % of DTPs would resume normal proliferation and were termed drug-tolerant expanded persisters (DTEPs). The authors were able to restrict the formation of DTPs/DTEPs through inhibition of the IGF-1 receptor, by inhibition of KDM5A demethylase, and through use of histone deacetylase (HDAC) inhibitors. This work has identified a chromatin-mediated reversible drug tolerant state in residual cancer populations that may account for pan-resistance.

## Epithelial–mesenchymal transition

A critical step in the process of invasion and metastasis is the phenotypic change in tumour cells known as EMT. Three types of EMT have been described and reviewed; type 1 EMT is associated with embryogenesis and development and type 2 EMT pertains to wound healing, tissue regeneration and organ fibrosis [72, 73]. Whilst these first two are affiliated with highly regulated physiological processes, type 3 EMT relates to the transformation of epithelial cancer cells in relation to the process of metastasis and is thus pathological [74]. It was reported by Boyer et al. [75] that the process of ‘cell scattering’, an essential step in invasion and metastasis, involves at least two biological events; (i) cell–cell dissociation as a result of the disruption of intercellular bonds and (ii) cell movement as a result of rearrangement of cytoskeletal proteins and the formation of new cell-substratum contacts. These events appear to occur simultaneously or synchronously within cells and lead to active cell migration. In normal cells there is a requirement for the activation of a range of highly controlled and spatio-temporally regulated signalling molecules to trigger the changes seen during EMT, which do not occur under normal circumstances. However, it is proposed that in cancer cells, oncogenic activation of signalling molecules may result in a cell-autonomous EMT process [75].

**Fig. 1:**
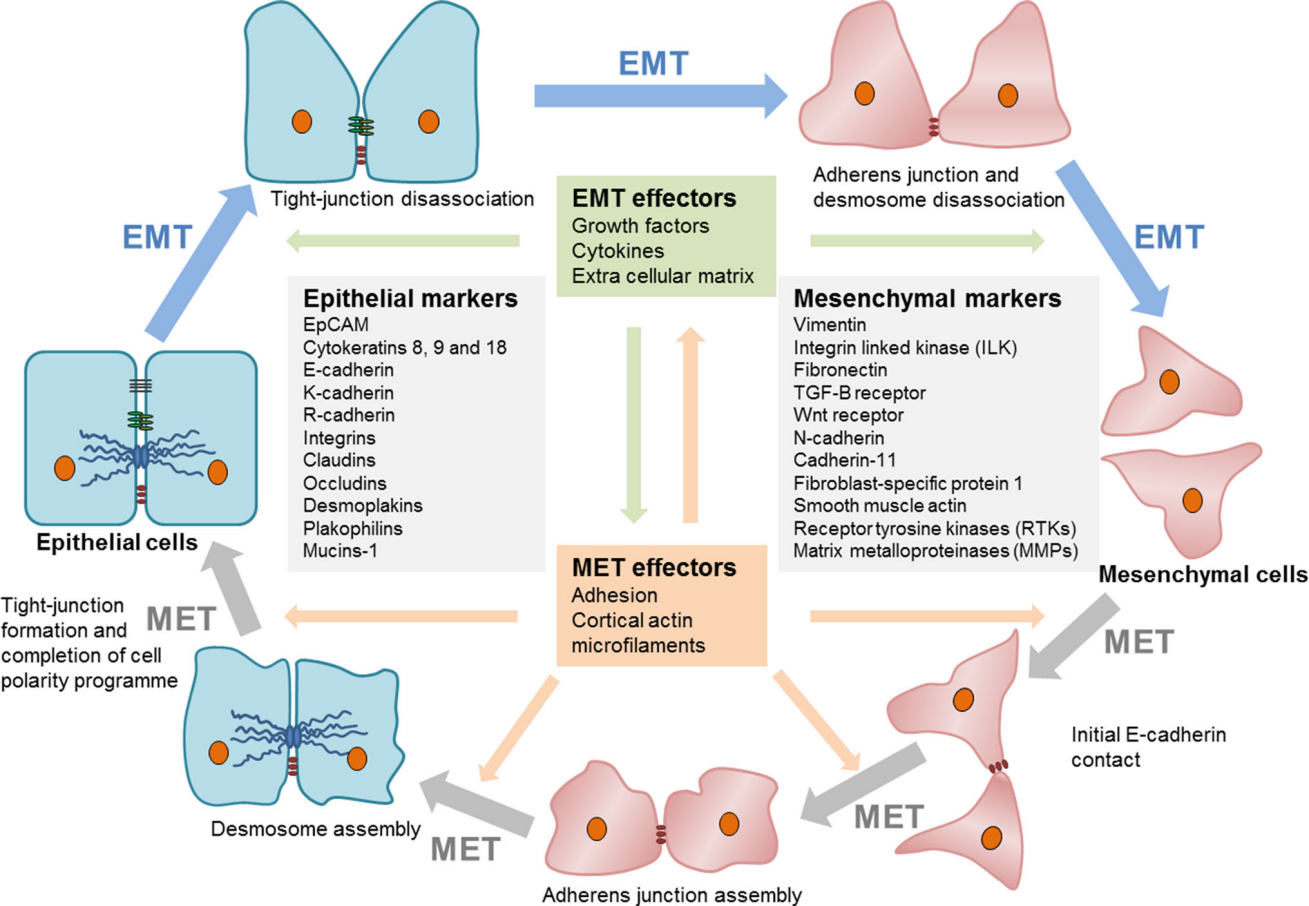
Epithelial–mesenchymal plasticity (modified from Thiery and Sleeman [77]); a representative diagram on the roles of epithelial–mesenchymal transition, mesenchymal–epithelial transition, and associated markers with respect to changes in cell morphology and behaviour

More than 95 % of primary breast cancers are of epithelial origin [76]. Epithelial cells have a cobblestone appearance and are firmly held in a relatively rigid structure through distinct contact between cells, constituted by tight junctions, adherens junctions, desmosomes and gap junctions (Fig. 1) [77]. E-cadherin is a widely studied transmembrane glycoprotein which in epithelial structures is essential for maintaining stable tissue architecture through cell–cell adhesion [78]. The intracellular domain of E-cadherin binds with catenins that in turn are linked to the actin cytoskeleton [79]. It is these interactions, as well as the homodimerisation occurring between the extracellular domains of E-cadherin molecules, which are essential for stable cell–cell structure [79]. Specific integrins also anchor epithelial cells to constituents of the extracellular matrix (ECM) such as laminins, fibronectin and collagen [80–82]. However, during EMT cell–cell adhesions involving E-cadherin in adherens junctions, occludins and claudins in tight junctions and desmoplakin in desmosomes, along with the affiliated apico-basal polarity, are lost as cells take on mesenchymal characteristics [73]. The cells become more elongated or ‘spindle-like’, flexible, mobile and therefore potentially invasive (Fig. 1) [77].

Mesenchymal cells have a less defined and structured organisation, lacking the apico-basal polarity of epithelial cells or a guiding basal lamina, and are thus subject to changes in the actin and intermediate filament cytoskeletal framework [72]. During EMT there is an increase in the secretion of proteolytic enzymes, which results in degradation of the ECM, mediated by matrix metalloproteinases (MMPs) and the urokinase plasminogen activator (uPA) system, thus enabling the cells to invade surrounding stroma and tissues [83]. It has been shown that activation of the uPA system is associated with a poor prognosis in breast cancer [84, 85]. More recently, the mesenchymal-like phenotype was also associated with decreases in sensitivity to current anti-cancer therapies including cytotoxic and molecular targeted agents [86]. The overall effect is a significant improvement in the metastatic efficiency of these cancer cells [87, 88].

In addition to the characteristic loss of expression of E-cadherin and epithelial-associated cytokeratins (CKs), EMT also involves an increase in the expression of vimentin, N-cadherin, the secretion of MMP enzymes and an accompanying increase in the expression of transcription factors (TFs) such as *TWIST*, *SNAIL* and snail homolog 2 (*SLUG*) that promote the mesenchymal phenotype. Korsching et al. [89] showed that vimentin expression correlated with tumour grade, the expression of alpha smooth muscle actin (ASMA) and also with the expression of genes including the epidermal growth factor receptor (*EGFR*), cytokeratin 5 (*CK5*) and cytokeratin 10 (*CK10*), which are all implicated in basal breast cancer. Despite questioning the degree to which EMT is responsible for the vimentin expression observed in invasive breast cancers, they concluded that vimentin expression was evidence of the final step of de-differentiation in tumours and was associated with invasion. The extent and nature of vimentin expression in breast cancer has previously been reviewed [90]. Sarrio et al. [91] have shown a relationship between a range of basal/mesenchymal markers and poorer outcome in breast cancers. Willipinski et al. [92] utilised a large series (>2200) of breast cancers to demonstrate that loss of CK and ectopic vimentin expression were significantly associated with a higher tumour grade, high mitotic index, and negative oestrogen/progesterone receptor (ER/PgR) status, and significantly related to clinical outcome in univariate analyses.

Dabbs et al. [93] recently revisited the use of E-cadherin immunohistochemistry (IHC) with respect to lobular breast cancers. The fact that lobular breast cancers either have poor E-cadherin expression or are completely devoid of it was confirmed in this work, yet histologically these tumours maintain an epithelial appearance. Moreover, an extensive screen of lobular breast cancer transcriptomes recently could not show any evidence for increased EMT in these cancers, despite the defining lack of the epithelial gate-keeper E-cadherin [94]. Alternative cadherins such as R-cadherin may be acting as the primary cell–cell adhesion mediator [95]. Additionally, it is apparent that the functional nature of a given cadherin varies depending on the biological context [96, 97]. Therefore, what constitutes a definitive EMT in cancer becomes individualistic, as it is clear that the classic EMT parameters do not always apply due to the likely presence of an intermediate or hybrid phenotype [98]. While the lack of E-cadherin may focally pre-dispose lobular breast cancer cells to EMT, it does not globally cause an EMT manifestation.

## Epithelial–mesenchymal transition in MRD

Given that research has concentrated increasingly on EMT in metastasis, the role of EMT in MRD has been a relatively recent focal point. Hence there have been a number of publications from various research groups reviewing aspects of EMT in CTCs/DTCs [99, 100]. The first paper assessing E-cadherin status in MRD appears to have been performed by Funke et al. [101] in 1996. This study used IHC staining for CK18 and E-cadherin in the bone marrow of breast and gastric carcinoma patients. They found individual CK18^+ve^ cells to be present as well as small clusters (2–9 cells) and larger clusters (>10 cells). Analysis of E-cadherin status revealed DTCs to be positive, negative, or heterogeneous in their E-cadherin expression. Specifically, breast DTCs tended to be heterogeneous with a relatively even number of E-cadherin positive and negative patients, whereas almost all analysed gastric carcinoma patients were negative with the exception of a single heterogeneous case. While there was no overall statistical indication of a trend for E-cadherin negativity in lone DTCs and positivity in DTC clusters, it was noted that 3 of 4 patients with homogeneous E-cadherin positivity had large DTC clusters in bone marrow. More recently, the number of original research articles attempting to test the EMT hypothesis in MRD is increasing. One of the earliest papers looking at EMT in CTCs/DTCs observed an EMT-like gene expression signature in breast cancer cells purified from pleural effusions using anti-epithelial cell adhesion molecule (*EpCAM*) antibody MOC-31-conjugated immuno-magnetic beads [36]. Microarray analysis was performed and revealed that the cells purified from pleural effusions were divided into two distinct subgroups that were termed ‘EP1’ and ‘EP2’. Some genes upregulated in the EP1 group were associated with the epithelial phenotype and included CKs, microtubule associated proteins, genes involved in cell–cell adhesion, cell survival and proliferation. In particular, the Ets-1 TF known to activate metastasis-associated molecules was selectively upregulated. Within the EP2 group genes associated with the promotion of an aggressive mesenchymal phenotype and EMT were upregulated and included MMPs, integrins, vimentin and *CXCR4*. The Willipinski et al. [92] study mentioned above also used protein-based assays to assess cell lines established from the DTCs of breast cancer patients. They found a loss of several epithelial-associated CK proteins as well as a gain of vimentin in these DTC cell lines when compared to MCF-7 and MTSV-1.7 reference cell lines [92]. The approach of utilising DTC cell lines for the analysis of the mesenchymal marker vimentin, among other markers, in MRD was originally published by Putz et al. [102] in 1999. This was expanded upon by a group that isolated DTCs from the bone marrow of breast cancer patients and using a PCR based assay found an upregulation of the E-cadherin transcriptional repressor *TWIST1* [103]. Another study assessing EMT in CTCs intentionally used negative selection only as their method of CTC purification and observed the overexpression of at least one of the following EMT promoting transcription factors; *TWIST1*, *SNAIL1*, *SLUG*, zinc finger E-box-binding homeobox 1 (*ZEB1*) and *FOXC2* in 15.4 % of breast cancer patients by qRT-PCR [104]. In the context of prostate cancer, CTCs were assessed by fluorescent in situ hybridisation (FISH) analysis and genomic imbalances in breast cancer gene 1 (*BRCA1*) were observed with a more advanced disease state. Further, these genetically aberrant CTCs were found to be vimentin positive by immunocytochemistry (ICC) staining [105]. Indeed, it has been shown that increased expression of vimentin in primary breast tumours is correlated with poor prognosis [106]. Additional studies have also reported on the expression of vimentin in human DTC cell lines [107] as well as in CTCs from clinical [41, 108] and mouse model blood samples [109].

More recently, published work attempting to assess EMT in CTCs/DTCs has expanded to include markers that are said to be typical of breast CSC-like cells (BCSC) [110–112]. These apparently mesenchymal BCSC are thought to be able to establish new distant tumour colonies as a result of their stem-like properties that enable the differentiation and generation of the various cell phenotypes observed in a heterogeneous tumour mass [113]. A couple of papers focusing on these aspects looked specifically at CTCs in early stage and MBC patient’s post-systemic therapy using PCR based assays [41, 114]. The first applied the commercially available AdnaTest and found an upregulation of the EMT promoting markers *TWIST1*, v-akt murine thymoma viral oncogene homolog 2 (*Akt2*) and phosphatidylinositide 3-kinase alpha (*PI3Kα*), in addition to the BCSC marker aldehyde dehydrogenase 1 (*ALDH1*) [114]. This work has been more recently complemented by Kallergi et al. [41] who demonstrated that *VEGF*, hypoxia-inducible factor 1-alpha (*HIF*-*1α*) and phosphorylated protein kinase B (*pAkt*) are expressed on CTCs in most MBC patients. Further, they showed that *TWIST* and vimentin are expressed on CTCs in both early stage and MBC [41]. Whilst *TWIST* downregulates the expression of E-cadherin, it also increases the transcription of the protein kinase B (*Akt*), which is known to inhibit apoptotic processes. It has been demonstrated that binding of *TWIST* to the *Akt* promoter and the upregulation of *Akt* results in resistance to paclitaxel [115]. An RT-PCR assay was further used to show that the proportion of *ALDH1* positive CTCs was higher in more advanced breast cancer patients and correlated with vimentin and fibronectin expression [116]. These gene expression profiles may have very important implications in clinical practice, where largely epithelial markers are used in the commercially available CTC assay devices that rely on marker detection. In an interesting published letter, it was outlined that in patients whose CTC numbers were assessed, a greater proportion of patients were positive for CTCs when their cancer was determined as either being *HER2* overexpressing or triple negative at diagnosis when compared to patients with luminal type cancer [117]. This may be reflective of breast cancer cell lines that are categorised into these respective molecular subtypes, as triple negative cell lines for example tend to fall into the Basal A and Basal B subgroups which frequently display mesenchymal and tumour initiating properties [118].

## Mesenchymal–epithelial transition

Whilst EMT/mesenchymal markers have been demonstrated on CTCs and DTCs, it has also been observed that human breast cancer metastases in liver, lung and brain often express higher levels of E-cadherin relative to the primary tumour and hence can be ‘more epithelial’ in nature [119, 120]. In addition to membranous E-cadherin promoting cell–cell adhesion, this epithelialisation has been attributed to the ability of the E-cadherin cytoplasmic domain to bind β-catenin, α-catenin and p120. This not only links the adhesion molecule to the actin cytoskeleton, but prevents nuclear localisation of β-catenin and in turn LEF-1/TCF mediated transcriptional activation of the Wnt signalling pathway [121], which has been demonstrated to promote an aggressive mesenchymal phenotype in cancer [122]. This phenomenon is also observed in other cancers and their metastases [123, 124], indicating that MET, may play an important role in the establishment of macrometastases. Indeed, it is suggested that the mesenchymal properties of CTCs and DTCs are insufficient for optimal malignant behaviour and in fact the ability to transition from an epithelial to a mesenchymal phenotype and then back to an epithelial state is an important determinant of aggressive metastatic behaviour [125]. Armstrong et al. report that the preponderance of MET events among lung metastases in rats bearing AT3 rat prostate adenocarcinoma tumours suggested an important functional relationship between the capacity to revert to a more epithelial state and metastatic growth in the lung parenchyma [126]. Recent studies have further illustrated the importance of MET in metastasis [127, 128] and others have shown that the EMP cycle can endow a unique gene expression profile on carcinoma cells, opening up new therapeutic avenues [129, 130]. The dynamic ability of cells to move across a spectrum of epithelial and mesenchymal states, as opposed to undergoing a one-way transition, is what we term epithelial–mesenchymal plasticity (EMP), an all-encompassing term which perhaps more accurately describes the variable nature of this axis in CTCs, DTCs, and the process of metastasis [131].

It is important to note that as implied by the concept of EMP, there exists intermediate states whereby cells can express both epithelial and mesenchymal markers to varying degrees and these are very likely to be more commonly observed than complete epithelial or mesenchymal states in the context of MRD. Some of the studies previously discussed have proposed the concept of an intermediary EMP phenotype in cancer, but the study conducted by Creighton et al. [56] was the first to visually demonstrate the phenomenon. While this study focused on residual breast tumour cells following treatment, the enrichment of mesenchymal and putative BCSC markers has also been observed in CTCs of breast cancer patients [132]. Recent work by Yu et al. [133] demonstrated both rare primary tumour cells and highly enriched CTC subpopulations that simultaneously expressed both epithelial and mesenchymal markers. They utilised a fluorescence RNA-ISH approach to quantify the proportion of epithelial (E^+^), mesenchymal (M^+^) and hybrid (E/M) tumour cells in; (i) epithelial and mesenchymal xenograft tumours grown in mice, (ii) tissue microarrays containing samples from both benign breast tissue and invasive primary breast cancers, (iii) CTCs isolated from the blood of MBC patients. One key observation of interest was that in patients responding to therapy, a greater proportion of CTCs switched to being E^+^ post-treatment, whilst in non-responders where disease progressed, the larger proportion of CTC switched to being M^+^ post-treatment.

This work has been supplemented by studies that have provided some of the first direct evidence of EMP by demonstrating the existence of EMP hybrids that harbour tumour initiating properties in CTCs purified from the blood of advanced prostate cancer patients [126] and patients with non-small cell lung carcinoma [108]. Moreover, Wu et al. [134] applied an RNA-ISH approach to CTCs isolated from a range of carcinoma patients and were able to subcategorise them as being either epithelial, mesenchymal, or hybrid. Based on the literature, studies are still underway attempting to further demonstrate the presence of the EMP hybrid cell, also described as metastable, and its coexistence with the BCSC phenotype in CTCs and DTCs [135, 136].

## Evidence that CTCs are malignant cells

In 2000, a paper published by Pretlow et al. [137] provided physical evidence that isolated CTCs could form tumours. This was the first study to describe the establishment of xenografts in nude mice from CTCs harvested from blood of 14 patients with advanced treatment-resilient metastatic cancer (11 prostate and 3 colon cancer patients). Lung metastases were observed to develop in mice that received CTCs from three of the patients (1 with colon and 2 with prostate cancer). Subsequent work has since been published illustrating that CTCs carry malignant characteristics. In 2002, Fehm et al. [44] examined ‘circulating epithelial cells’ (CEC) or ‘circulating epithelial tumour cells’ (CETC), to determine whether they were aneusomic—contained aberrant chromosome copy numbers relative to the normal diploid human cell—and compared these aneusomic patterns to those from the matched primary tumour. The group was able to match the aneusomic pattern of CEC and primary tumour touch samples in 10 of 13 patients, also noting gains of chromosomes were more frequent than losses. In some cases the pattern of aneusomy differed between CEC and primary tumour, reasons proposed for this including; (i) shed CEC undergo further genetic modifications, (ii) overlapping signals if more than two copies are present, (iii) analysis in touch preparations may have missed cells with matching aneusomic patterns and (iv) the CEC were shed from a metastasis and not the primary tumour. Additionally, further studies have demonstrated the cancerous nature of CTCs, for example in breast cancer patients that were found to overexpress the proto-oncogene *HER2* [138]. Epidermal growth factor receptor, known to be frequently overexpressed in breast cancers and correlated with a poor prognosis, has also been detected on CTCs in 38 and 44 % of early and MBC patients, respectively [139]. The tumourigenic and metastatic potential of CTCs has been recently further demonstrated and expanded upon using xenograft systems. Baccelli et al. [140] successfully grafted CTCs from breast cancer patients into the femoral medullary cavity of non obese diabetic-severe combined immunodeficiency gamma (NSG) mice—NSG mice lack mature lymphocytes, NK cells, and have several compromised cytokine signalling pathways in addition to an impaired innate response. They noted that greater success was achieved when the isolated CTCs were subject to FACS sorting for EpCAM, CD44, CD47, and the proto-oncogene MET (a HGF receptor tyrosine kinase). Whilst not all sorted CTCs were positive for MET, the metastases that grew in mice were MET-enriched. Hodgkinson et al. [141] were able to graft small cell lung cancer CTCs following subcutaneous injection into the flanks of NSG mice, and then confirmed that these tumours were derived from the isolated CTCs through comparison of genomic profiles for *TP53* and *RB1*. Yu et al. [142] isolated CTCs from breast cancer patients and were able to establish cells lines using non-adherent in vitro culture, where it was noted that greater success of cell line formation occurred using CTCs from patients who were therapy-resistant. Successful xenografts were subsequently established from a portion of these cell lines in NSG mice. Interestingly, Yu et al. [142] utilised these CTC lines for the screening of mutations that would enable targeting of drug sensitive pathways, of particular interest were those that differed between the primary tumour and CTCs, thus emphasising the importance of continual CTC monitoring.

## Cancer dissemination and dormancy

Is cancer dissemination an early event during tumourigenesis? The prevailing belief is that cancer arises from an accumulation of genetic and epigenetic changes over time, leading to cellular disorganisation and uncontrolled growth, and that invasiveness only arises late in the process after further changes. However emerging research is revealing cancer dissemination can occur early in cancer development [37, 143]. In mouse models, systemic dissemination of tumour cells has been shown to occur after early epithelial changes in the mammary gland [37]. Moreover, in both mouse and human, the number of disseminated cells appears to be unrelated to the size of the primary tumour [37, 144]. Research by Husemann et al. [37] using two transgenic mouse models (*HER2* transgenic mouse model—BALB-NeuT, and mouse mammary tumour virus-polyoma middle T-antigen transgenic mouse model—MMTV-PyMT) demonstrated that dissemination of tumour cells can occur in pre-invasive stages of tumour progression and that the number and genotype of seeded tumour cells were not associated with tumour size. The highest dissemination rates have been shown relative to the total number of cancer cells in the primary tumour to occur early after transformation. Interestingly, disruption of the basement membrane underlying hyperplastic epithelia was observed in the BALB-NeuT system. This was attributed to activation of proteolytic systems in breast epithelia and found to be associated with young age and atypical ductal hyperplasia (ADH) following cDNA array analysis of microdissected samples [37]. However, electron microscopy carried out on both transgenic models, revealed that epithelial cells were crossing the basement membrane prior to any indication of basement membrane degradation. In addition, HER2^+^ DTCs detected in the lung and bone marrow of mice with ADH (a pre-malignant breast condition which is not invasive), were confirmed to be malignant and by comparative genomic hybridisation to have arisen from the primary lesion [37].

In recent years it has also been demonstrated that disseminated cancer cells often display a far less progressed genomic state than would be expected if this original perception of cancer metastasis were the case [145]. In a review by Klein [145] the late dissemination model was found to be incongruent with the timing of metastases relative to their tumour of origin even when tumour volume doubling time of primary tumour vs each metastases was taken into consideration. Vogelstein et al. [146] speculated in a review that perhaps there are no metastasis genes and therefore the need for genetic and epigenetic aberrations to accumulate over time is a void concept. It was suggested that it may be a purely stochastic process depending on which cells leave the tumour, where they go and when. This statement is based on work demonstrating that normal cells have been shown to survive and grow with functional vasculature on lymph nodes, a common site of metastasis. This work is complemented in a similar study by Podsypanina et al. [147] that demonstrated the ability of normal mammary epithelial cells to survive in ectopic sites following injection into the circulation of mice and following induced oncogene expression, to be able to multiply and colonise the new sites. Klein subsequently reviewed this publication and goes on to emphasise that the work highlights more than early vs late dissemination, but specifically whether malignant cells seen in a metastasis evolve inside or outside the original tumour mass. The capacity for early dissemination and implications of EMT in this were highlighted in a recent study from Rhim et al. [148], where fluorescence lineage labelling revealed the spread of pancreatic cancer cells prior to any histological or clinical indications of a primary malignancy.

Dormancy of cancer cells that disseminated early from primary tumour is thought to be the reason behind the recurrence of cancer in individuals years or decades after a successful primary tumour removal. Dormant cancer cells may exist as quiescent solitary cells [149] or micrometastases in a state of balanced proliferation [150] which are not clinically apparent [151]. Like their DTC counterpart, residual primary tumour cells are thought to employ dormancy via similar mechanisms; quiescence, EMT, and CSCs [152], all of which are discussed in this review. Dormancy in the context of primary disease is likely to be as a consequence of therapeutic intervention and may indeed also apply to DTCs and micrometastases. In the instance where treatment has yet to be initiated and occult distant disease already exists, the driving cause of dormancy is different—overcoming foreign microenvironment pressure [153]. Schmidt-Kittler et al. [154] demonstrated that disseminated cells found in the bone marrow of non-MBC patients harboured fewer and different genomic aberrations than the primary tumour and suggested that these seed cells must have spread before surgery or even before first diagnosis. They go on to suggest that the relatively small number of chromosomal aberrations in many patients without overt metastases, but with disseminated cells, may point to a deceleration of the carcinogenic process in these cells, perhaps due to environmental constraints, which may account for dormancy. The length of the period of dormancy may therefore reflect the time necessary to accumulate further aberrations required for unrestrained growth. These aberrations may differ substantially to those in the primary tumour, accounting for the observed genetic differences seen between metastatic and primary tumours [155]. There is evidence however that the genetic profile of primary breast cancer can resemble that of metastases [156], and this has been observed in other types of cancer [157]. This suggests the early and late independent progression models are not common and that the capacity to metastasise either is or is not imprinted within an entire tumour. How the relationship between dormancy and mutation is modified in the clinical situation with systemic treatments and treatment resistance is yet to be determined, although some light has been shed on shifts in circulating DNA patterns during the course of treatment and site of distant metastasis [158].

## CTCs/DTCs and ductal carcinoma in situ (DCIS)

Ductal carcinoma in situ (DCIS) is a non-invasive form of breast cancer that involves the neoplastic proliferation of tumour cells within the ductal-lobular structures of the breast that have not invaded the ductal basement membrane. Diagnosis of DCIS has increased dramatically in recent years as a result of increased mammographic screening and now represents up to 20 % of breast cancer cases [159]. Although DCIS is classified on the basis of histopathological criteria and growth dimensions/prognosis may to some extent be predicted on this basis, much remains to be understood regarding the behaviour of DCIS. Whilst it has been widely assumed to have no metastatic potential, patients are considered to have a relatively increased risk of progression to invasive breast cancer and of local recurrence particularly if the DCIS is of high nuclear grade and overexpresses *HER2* [159, 160]. Perhaps surprisingly, up to 3 % of DCIS patients are found to have axillary lymph node metastases [161]. This observation coincides with the work by Husemann et al. [37], as discussed earlier, who demonstrated in mouse models that tumour cells can disseminate from even the earliest epithelial alterations, and so it appears that this may be the case in at least some human DCIS patients. Sentinel node involvement has been routinely reported in between 1 and 13 % of DCIS patients without evidence of invasion at the primary site (although this of course could just be that the invasive disease was not apparent in the sections reviewed by the histopathologist). In a study of 266 patients diagnosed with DCIS, Banys et al. [162] identified DTCs in bone marrow aspirates of 34 (13 %) patients. They found no statistical correlation between the presence of DTCs and clinicopathological features. Interestingly, whilst 3 of the 221 patients who underwent sentinel lymph node biopsy were positive for tumour cells in the sentinel node, none of these had DTCs in the bone marrow. However, a smaller study by Sanger et al. [160] found that 4 of 19 (21.1 %) patients with pure DCIS had detectable DTCs in their bone marrow. Notably, they stress the need for the use of appropriate antibodies to detect all DTCs, including those that may have undergone an EMT and are not detectable by commonly used CK antibodies. They propose that the 13 % DTC detection rate reported by Husemann et al. [37] may be misleadingly low due to the antibody used. Banys et al. [162] also used the same CK antibodies, which may potentially explain their similar findings. The clinical weight of these early metastatic events in the context of DCIS is still ambiguous. While sentinel lymph node metastasis is detected in 3–4 % of DCIS patients, the rate of overt distant metastasis in the patients is low [163]. Further, given that tumour stage is an independent negative prognostic indicator [164], the rate of effective dissemination in early lesions is unclear. Thus the role of MRD detection in predicting the prognosis of DCIS patients is still of unknown clinical significance but is an exciting area of future translational study.

## MRD and the CSC phenotype

CSCs were first described in the context of leukaemia, when Bonnet et al. [165] reported that only a minor subset of leukemic cells with the *CD34*^+^*CD38*^−^ cell surface marker profile, when transplanted into SCID mice, resulted in dissemination and survival with leukemic cell morphology similar to that seen in the donor patient. Putative CSCs have since been identified in a range of solid tumours, including; breast, colon, brain and prostate [110, 166–168]. In studies of MRD it is known that CTC and DTC populations are very heterogeneous, even within the same patient, for example morphologically [169] as well as molecularly [170]. This is also true of cancer cells within the primary tumour, as alluded to earlier in this review. There is growing evidence suggesting that only certain subpopulations of primary tumour cells acquire the characteristics necessary to break away from the primary site and enter the circulation and it is increasingly proposed that only certain subpopulations of ‘aggressive’ CTCs/DTCs are able to progress into metastases. Along these lines, CTCs capable of short term culture were found to better predict outcome than patients without culturable CTCs [133]. The concept of CSCs implies that there is a small population of cells within a primary tumour that have the propensity to be tumourigenic and multipotent, hence CSCs provide a possible explanation as to what this subpopulation of cells is comprised of and how it has the capacity to propagate cancer progression.

In breast cancer these putative CSCs were originally identified by Al-Hajj et al. as *CD44*^+^*CD24*^−/low^ through the use of xenotransplantation experiments [110]. Studies indicate they make up approximately 10–20 % of tumour cells in the primary mass, although the degree of variation is extensive [171, 172]. *CD44* is a cell adhesion molecule that has been associated with stem cells in normal breast tissue but is also found on many other cell types. It has been reported that *CD44* potentiates the adhesion of breast cancer cells to endothelial cells in the bone marrow [173], thus possibly mediating bone-specific metastasis. *CD24* is normally expressed during the early stages of B cell development and is not usually expressed in adult human tissues but has been demonstrated in human cancers. In breast cancer cell lines, *CD24* expression reduces stromal cell-derived factor-1-mediated migration and signalling via *CXCR4*, suppressing their metastatic potential, whilst *CD24*^−/low^ cells have conversely been shown to increase metastatic potential [171]. In subsequent studies, an additional candidate marker for the CSC phenotype has been utilised—*ALDH1*, which was originally identified in the context of retinoblastoma [174], and is involved in the oxidation of intracellular aldehydes and is highly expressed in many stem and progenitor cells [111, 113, 175]. This marker has since been incorporated in methods used for the detection and assessment of MRD both molecularly [114, 116] and visually [176, 177].

There is a substantial body of work indicating that EMP plays a role in the development of cancer cells harbouring traits that define a CSC [113, 178–180]. Thus there is mounting focus on and evidence for the possibility that CSC subpopulations of either the primary tumour or CTCs/DTCs, at least partly through EMP, acquire CSC attributes such as quiescence, self-renewal, asymmetric division and multi-drug resistance [56, 114, 116, 132], as well as an inherent resistance to radiation [181]. These characteristics allow the cells not only to survive in hostile environments such as the circulatory system and bone marrow, but to also survive conventional therapies and subsequently drive tumour growth and the establishment of metastases. It is important to acknowledge that the CSC concept is still under much debate [182, 183]. Work from the Weinberg lab showed that BCSC could be derived from differentiated mammary cells [184], whilst Liu et al. [185] revealed that the CD24^−^/CD44^hi^ subpopulation was distinct from the ALDH1^+^ subpopulation in BCSCs, thus questioning the use of these together to define the putative BCSC phenotype. Moreover, Sarrio et al. [186] demonstrated stem-like attributes in normal-like breast cancer cell lines independent of mesenchymal state. The consequence of studies such as these is that the complexity of the relationship between EMP and BCSC is either greater than current comprehension, or that its validity is questionable. Particularly interesting articles have called into question the validity of xenotransplantation as a measure of tumourigenicity when investigating these CSCs [187, 188], discussing whether these cells really are responsible for tumour propagation is questioned as examples of leukaemia are given where cells not sorted for CSC markers are still able to form tumours from low starting numbers. There is concern over this point of argument as it compares findings in solid carcinomas to systems using non-solid cancers. However, similar work demonstrating the tumourigenic capacity of melanoma cells using low numbers of unsorted cells in NSG mice has also been published [189, 190]. Despite all this, the key observation that has remained consistent is that these particular ‘CSC’ subpopulations, whether or not they are actually stem-like, are substantially more aggressive in that they persist after treatment and have a greater tumourigenic capacity.

## Characterisation of CTCs/DTCs

CTCs are typically found in extremely low frequencies, being as few as 1 per 10^9^ blood cells or 1 per 10^6^–10^7^ mononuclear cells [191–193]. However, CTCs/DTCs are often much larger than blood cells, with the mean diameter of tumour cells in the blood of breast cancer patients reported as 29.8–33.9 µm [194], whilst the vast majority of blood leukocytes are 8–12 µm. According to one publication, the basic morphological criteria for CTC include a nucleus larger than 16 µm, irregularity of the nuclear contour, the presence of visible cytoplasm and a nuclear-to-cytoplasmic (N/C) ratio greater than 0.8 [192]. Whilst some of these criteria are commonly shared by other studies, it is worth noting that the exact cut-off values can vary, and alternative criteria such as anisonucleosis may be utilised [195]. DTCs share these characteristics, as well as being reported to have a tendency to occur in cell clusters, with strong or irregular cytoplasmic CK staining, visible CK filaments, a large nucleolus and often a granular or stippled nucleus [196, 197]. Having described these morphological characteristics, one group has published work related to CTCs displaying morphological features heterogeneous in nature. CTCs were observed to have an N/C ratio that spanned from high to low and that their overall size varied from being larger to smaller than white blood cells (WBC). Further comparisons were made between the morphology of CTCs and primary/metastatic tumour from the same patient. A key conclusion was that CTCs maintain primary tumour cytology characteristics and are representative of the heterogeneous nature of the primary/metastatic tumours. Their results argued against the hypothesis that only particular subsets of carcinoma cells have the capacity to disseminate, such as CSCs [169, 198].

In 2007, the American Society of Clinical Oncology (ASCO) breast cancer consensus panel assessed the use of prospective markers in breast cancer for various purposes. Some of the approved included; Cancer antigen 15-3 (CA 15-3) and Cancer antigen 27.29 (CA 27.29) kits that measure Mucin 1 (*MUC1*) levels in peripheral blood, assessment of carcino-embryonic antigen (*CEA*) levels in blood and *ER*, *PgR*, *HER2* status of the primary tumour. Markers that lacked sufficient evidence for approval included DNA/ploidy via flow cytometry, p53 and the presence/absence of CTCs/DTCs [199]. The promise behind CTCs/DTCs with respect to their use in the clinic is that they provide a potential source of repetitive low-invasive ‘liquid’ biopsies that allows for continual ‘real-time’ monitoring of cancer patients that are at risk of relapse [200]. The reasoning behind the decision on CTCs/DTCs by the ASCO was the observation that not every patient found to be positive for these cells would necessarily relapse as expected. Several interventional trials have been established to measure the clinical utility of CTC enumeration [201], and despite confirming a strong prognostic relationship between CTC burden and therapeutic response, the SWOG S0500 trial could not demonstrate clinical utility in the form of increased overall survival (OS) or progression free survival (PFS) in patients who were put on alternative chemotherapeutic regimens due to persistent or increased CTC counts following initial therapy [202]. The more intensive CirCe01 trial is ongoing, and interim analysis has recently confirmed the strong prognostic power of CTCs [203]. Consequently, there has been an increasing amount of work attempting to characterise CTCs/DTCs molecularly and phenotypically in order to identify MRD that poses a true threat by gaining an understanding of the underlying biology. As a result, one key observation, which has been repeatedly shown, is that CTCs/DTCs are very heterogeneous. Efforts to characterise MRD are greatly hampered, not only by scarcity, but by the variety and specificity of the various enrichment and detection methods employed by different laboratories. This aspect has been previously reviewed in a number of articles [204–208].

As EMP may be an essential process in the generation and function of CTCs and DTCs, multiple publications have attempted to assess EMP-related markers both molecularly and phenotypically, as discussed earlier. It may partly be through this very process that the large variation in the presence of molecular markers arises and can be seen on CTCs/DTCs. Additionally, if the notion that EMP has a critical role in MRD holds true, then a number of technical implications could well be responsible for variation in results as alluded to earlier. The key ramification relates to the fact that to date a large proportion of enrichment and detection methods have relied on the expression of epithelial markers, principally *EpCAM* and CKs such as *CK8*, *CK18* and *CK19*. In fact this has been the case for decades, for example some of the earliest work utilising EpCAM and CK18 as a means of detecting DTCs was performed by Kubuschok et al. [209] in 1999 and Schlimok et al. [210] in 1987. Therefore, because these can be downregulated or lost during EMT, there is potentially a bias in the molecular and phenotypic characteristics reported thus far. It has certainly been demonstrated that some subtypes of breast cancer are under-represented in MRD characterisation studies, again due to lack of epithelial markers [211]. One way that groups have attempted to circumvent this problem is to employ multi-marker approaches both in the enrichment step and the detection process [212–218].

These studies, and many others, have highlighted the variations in molecular and phenotypic expression of their selected markers between individual CTCs/DTCs, even within the same patient. For example, the study performed by Strati et al. [219] demonstrated high heterogeneity in CTC gene expression in both early breast cancer (EBC) and MBC. They found that in EBC patients nearly half were positive for *CK19* and *TWIST1*, whilst approximately 20 % or less were positive for melanoma-associated antigen 3 (*MAGE*-*A3*), *HER2*, human telomerase reverse transcriptase (*hTERT*) and human mammaglobin (*hMAM*). When they looked in MBC patients, roughly 50 % were positive for *CK19*, 40 % for *TWIST1*, 30 % for *hMAM* and 20 % or less for *MAGE*-*A3*, *HER2* and *hTERT*. Consequently, because this diversity in MRD has been repeatedly witnessed, a new mindset on how to investigate these cells has evolved. As a result, methods embracing approaches towards MRD that capture the individual cell, specifically in regards to molecular analyses, are being utilised. To our knowledge, Powel et al. [170] was the first group to isolate single CTCs from breast cancer patients for subsequent downstream analysis with a high throughput molecular assay. Sixty-five blood samples from 50 breast cancer patients, both EBC and MBC, were subjected to an in-house CTC enrichment device, the MagSweeper. They isolated 510 cells with a pipette guided by microscopy and the RNA from each subjected to a nested RT-qPCR approach using the Fluidigm system. Of the 510 cells, 105 were confirmed as CTCs according to their set parameters, and of the 87 genes assessed only 31 were considered ‘consistently’ detectable in at least 15 % of analysed CTCs. Two major subgroups of expression were identified irrespective of patient, and neither of which was consistent with the profiles of different breast cancer cell line subgroups. This study provides an indication of the extent of variation occurring between CTCs of different patients as well as within the same patient, in addition to the sparse nature of expression of some molecular markers in MRD.

Early studies focusing on DTCs have provided insight into characteristics of functional benefit such as the downregulation of major histocompatibility complex (MHC) class I expression, which has been suggested to provide an advantage by evading cytotoxic T-cell mediated cell death. Using an IHC approach, pioneering work by Pantel et al. [220] identified isolated tumour cells within the bone marrow of both breast and stomach/colon cancer patients lacking MHC I expression. This was noted to be more frequent in the breast cases as opposed to the stomach/colon cases and attributed to the tendency of breast cancers to preferentially develop overt metastases in bone. It was also noted that a mixture of MHC class I positivity and negativity was seen within 13 % of patients, thus reinforcing the heterogeneous nature of these cells. A statistically significant correlation between incidence of MHC I negative DTCs and a poorly differentiated primary tumour was observed and also confirmed in a separate study [221]. This latter study also demonstrated a poorer prognostic outcome in patients with MHC I negative DTCs. Similar work investigating uPAR expression in individual DTCs of breast cancer patients has also been performed following the original work by Heiss et al. [222] in gastric carcinoma. In addition to ICC, Pierga et al. [223] isolated EpCAM positive DTCs and subjected them to qRT-PCR for relative expression analysis of uPAR mRNA. They found that while not all patients had uPAR+ DTCs, of those that did, half had high uPAR mRNA levels. These DTCs with high uPAR expression significantly correlated with respect to their presence in patients with more aggressive cancers, for example 5 out of 6 patients with HER2+ cancer had high levels of uPAR in their DTCs. The association between uPAR+ DTCs in patients with aggressive primary cancer has also been observed in the context of prostate carcinoma [224]. In addition to the known tissue remodelling function of plasminogen activation via uPAR, alternative pathway activation (e.g. ERK/MAPK, HER2, FAK, Src) also contributes to metastasis-promoting phenotypes such as cell division and migration [225], thus providing a reasonable explanation for the observations of uPAR in DTCs. Work that first assessed ICAM-1 expression by DTCs was performed by Passlick et al. [226] who found that most NSCLC patients with an ICAM-1^−ve^ tumour had DTCs that remained ICAM-1^−ve^, while a larger proportion of patients with ICAM-1^+ve^ tumours switched to ICAM^−ve^ DTCs, and that it was the patients with ICAM-1^−ve^ DTCs that tended to have worse outcome. However, it was the work by Tsujisaki et al. [227] describing elevated serum ICAM-1 levels in malignant cancer patients that set the precedent for the study directly assessing ICAM-1 status on DTCs. Passlick et al. [228] then performed a correlative study assessing MHC I and ICAM-1 status in NSCLC tumours in relation to lymph node metastasis and bone marrow DTC positivity. It was found that reduced/absent MHC I expression correlated significantly with lymph node metastases as did a lack of ICAM-1 expression. The lack of ICAM-1 expression provides an interesting mechanism of survival by reducing monocyte and T-cell mediated destruction. However, this particular study did not directly assess ICAM-1 status in either the lymph node metastases or the DTCs, and no correlation was found between MHC I/ICAM-1 negativity and DTC positivity in bone marrow. Surprisingly, even though a role for ICAM-1 in DTCs has been described and considered for some time, there is still a lack of work directly assessing its expression in the context of metastasis and prognostic outcome across a range of carcinomas.

Recent work has turned to applying higher throughput genetic techniques to DTCs in order to collectively identify potentially key genomic alterations, but just as importantly, to demonstrate the applicability of such techniques to DTCs and therefore the prospect of using said methods as part of routine analysis of MRD. Holcomb et al. [229] applied an array comparative genomic hybridisation (aCGH) technique to DNA from small numbers of 10-20 DTCs isolated from the bone marrow of prostate cancer patients and identified losses in 8p23, 10q, 13q, and 16q, while copy number gains were seen in 8q. While the authors describe these changes as typical of prostate cancer, some affected genes such as CDH8 and CDH11 provide interesting avenues of investigation across a range of carcinomas. This aCGH method was further refined in a subsequent study comparing copy number variations (CNVs) in primary breast cancer to matched single DTCs using higher resolution single cell aCGH [230]. CNVs were observed to correlate between the primary tumour and DTCs, including losses at 8p, 11q, and gains at 1q, 8q and 17q. The results of this study appear to support the sequential clonal evolution theory of cancer, and yet similar work in breast cancer revealed discordant CNVs between matched primary cancer and DTCs [231], suggestive of the parallel progression/CSC model. Moller et al. [232] expanded on this approach by the inclusion of single cell next generation sequencing, thereby enabling not only CNV detection by aCGH, but also the ability to confirm CNVs and the capacity to detect copy-neutral loss of heterozygosity (cnLOH). Analysis was carried out on two cases of matched primary breast cancer and DTCs; the first revealed highly concordant genomic aberrations between tumour and DTC including monosomy 4, deletions on chromosomes 6, 16, 17, and duplications on chromosomes 1 and 17. The second on the other hand revealed a number of observations in DTC that were either sub-clonal in the tumour or absent altogether, such as a gain in 16p that was sub-clonal, trisomy 21 that was absent in the primary, and cnLOH of an allele on chromosome 13 that was present as a sub-clonal deletion in the tumour. Despite the mixed picture seen in the results of investigations into the behaviour of CTCs/DTCs, some overlapping data has provided insight into the potential importance of EMP, CSCs and the mechanisms they potentially help regulate such as; motility, invasion, therapy resistance, dormancy and cell survival by proliferation, modulation of senescence, or possibly anti-apoptosis.

## Oestrogen and progesterone receptor status in MRD

It is currently standard practice to determine the pathological grade and stage of the primary tumour and to examine HRs including ER alpha (ERα) and PgR using IHC. Tumours that are identified as HR positive have been shown to have a better prognosis in terms of overall survival, while HR negative tumours appear to have a more aggressive phenotype [233, 234]. Moreover, the presence of HRs in cancers provides a therapeutic target and allows the use of anti-oestrogen drugs improving survival for women with these tumours. However, many *ERα*^+^ tumours develop either de novo or acquired hormone resistance and thus progress to metastases even with anti-endocrine therapies. It has long been observed that the *ERα* and/or *PgR* status of the metastatic tissue may be discordant with that of the primary tumour [235–237], with *ERα*^+^ but *PgR*^−^ breast tumours comprising the poorer-prognosis Luminal B subtype.

Concordance rates between the primary and metastatic tissue have been found to be as low as 46 % and both the gain and loss of each HR has been demonstrated in metastases relative to the primary tumour [237–239]. The therapeutic impact of HR discordance was illustrated in a recent publication, where it was observed that HR^+^ patients who went on to relapse with HR^−^ metastases have significantly worse outcome [240]. Interestingly, patients that are HR^−^ at primary diagnosis but relapse as HR^+^ have better outcome than patients who have a HR^−^ recurrence irrespective of their original primary tumour HR status [241]. This and multiple other studies have led to speculation that patients with HR^+^ metastases, who had a HR^−^ primary tumour, may benefit from endocrine therapy, but as yet there has been no prospective randomised trial analysing the impact on survival of biopsy-driven treatment [242]. There have been some discrepancies in the reported rate of gain or loss of *ERα* and *PgR* expression in metastases relative to the primary tumour. Amir et al. [243] report that in a study on MBC patients, 12.4 % patients with an *ERα*^+^ primary tumour had *ERα*^−^ metastases, whilst 13.2 % of patients with an *ERα*^−^ primary tumour had *ERα*^+^ metastases, indicating a similar level of gain or loss of the oestrogen receptor. However, in the same patient group, 42.7 % of *PgR*^+^ primary tumour patients had *PgR*^−^ metastases, whilst 16.0 % of *PgR*^−^ primary tumour patients had *PgR*^+^ metastases, indicating a greater loss than gain of *PgR* expression in metastatic tissue. Nishimura et al. [244] report a drop in both *ERα* and *PgR* expression from primary to metastatic tissue, with *ERα* and *PgR* levels dropping from 63.9 to 56.7 % respectively in primary tumour, to 57.7 and 43.3 % respectively in metastases. The implication of this work as a whole is the potential for repeated biopsies in order to individually manage a patient’s therapy regimen by either giving the option to partake in a treatment which may be beneficial, or to withdraw from a treatment plan that is doing more harm than good. However, major limitations of assessing HR status in this manner relate to cost, time and invasiveness and so MRD has become the potential alternative source of information in this regard.

Given the role of CTCs/DTCs in the progression to metastases, and their persistence in patients after surgery and adjuvant therapy, it is unsurprising that the HR status of CTCs/DTCs has also been investigated and found in many instances to differ to that of the primary tumour. What may be surprising is that the discordance between primary tumour and CTCs HR status appears to be much greater than that seen between primary and metastatic tissue. Aktas et al. [114] found that in 87 CTC^+^ MBC patients, 77 % of those with *ERα*^+^ tumours had *ERα*^−^ CTCs and 87 % of those with *PgR*^+^ tumours had *PgR*^−^ CTCs. Concordance rates for *ERα* and *PgR* were 41 and 45 % respectively, and they observed that most CTCs were *ERα*^−^ and *PgR*^−^ (81 and 90 % respectively). A separate study by Fehm et al. [245], examining CTCs in primary breast cancer patients, found concordance rates between *ERα* and *PgR* status of CTCs and primary tumour to be 29 and 25 % respectively. Whilst DTCs were also isolated and detected in this study by IHC targeting epithelial CKs, no subsequent analysis of *ERα* or *PgR* status was performed on the DTCs. Another study assessing CTCs in MBC patients during the course of therapy also compared HR status of CTCs to patient pathology data. They found that 45 % of patients with *ERα*^+^ primary tumours had *ERα*^−^ CTCs, whilst 78 % of patients with *PgR*^+^ primary tumours had *PgR*^−^ CTCs [246]. Nadal et al. [247] expanded on these preceding studies by investigating HR status in CTCs compared to primary tumours and found that discordance was once again present, in this instance at greater proportions with respect to *PgR*. Perhaps their most intriguing finding however related to the observation that heterogeneity in HR status was seen within individual patients who bore both HR^+^ and HR^−^ CTCs.

When examining DTCs in primary breast cancer patients, Fehm et al. [248] found that there was a concordance rate of 28 % between the *ERα* status of the primary tumour and DTCs. Despite 88 % of patients having an *ERα*^+^ primary tumour, only 12 % of DTCs overall were *ERα*^+^, and as was the case with CTCs in the study by Nadal et al. [247], *ERα* expression was determined to be heterogeneous in 10 of 38 (26 %) patients with more than one DTC. They reported only one instance where a patient had an *ERα*^−^ primary tumour but *ERα*^+^ DTCs. This discordance between DTCs and primary tumour had been previously illustrated in a study by Ditsch et al. [249], who found that only 18 % of patients with *ERα*^+^ primary cancer had *ERα*^+^ DTCs. It is suggested by studies with such findings that the lack of *ERα* expression on CTCs and DTCs may be due to the clonal heterogeneity of the primary tumour [245, 248], in addition to the more aggressive and invasive features of *ERα*^−^ cells [250, 251].

Patients with *ERα*^−^ primary tumours typically have a worse prognosis than patients with *ERα*^+^ tumours and thus it may be that within heterogeneous tumours, the more aggressive and invasive *ERα*^−^ cells have increased likelihood of dissemination. As discussed earlier, it has been proposed that subpopulations of MRD with tumour initiating properties share characteristics reflective of an EMT signature and may be CSCs. In support of this, it has been demonstrated that a loss of *ERα* expression coincides with and may induce, EMT. Oestrogen receptor silencing of the *ERα*^+^ non-invasive MCF7 breast cancer cell line via siRNA resulted in oestrogen/tamoxifen-resistant cells that had altered morphology, increased motility, a switch from a CK to a vimentin-based cytoskeleton and increased invasive properties [252, 253]. It was also noted that key transcriptional factors that drive EMT were upregulated in these cells. Guttilla et al. [254] were able to demonstrate the loss of *ERα* in MCF7 cells that were subject to prolonged 3D culture conditions, as upon returning the cells to standard 2D culture, they were enriched in EMT and CSC characteristics. These studies coincide with work suggesting that *ER*α can stimulate *GATA3* expression and drive *FOXA1* co-expression, both of which oppose EMT [255], and work from our own lab showing that the transcription factor c-Myb, which mediates the pro-proliferative effects of oestrogen in breast cancer cells, has a reciprocal, inverse role on suppressing the EMT driver Zeb1, and is itself transcriptionally suppressed by Zeb1 [256]. Therefore a potential role for the silencing of *ERα* exists in the invasive and metastatic processes, in turn providing a lead as to which CTCs/DTCs might be of greater concern. Unfortunately, to date there appears to be no studies published that directly compare *ERα* and *PgR* status on primary and metastatic tissue, as well as CTCs and DTCs. Results from studies such as those mentioned above would indicate that whilst *ERα* and *PgR* expression seems to be largely lost in CTCs and DTCs, it is regained to some extent in metastatic tissue.

## HER2 status: differences in primary tumour, MRD and metastases

Amplification of the growth factor receptor *HER2* gene and subsequent overexpression of *HER2* occurs in approximately 12 % of primary breast cancers. Tumour cells that are *HER2*^+^ have an advanced extravasative potential that results in an aggressive form of the disease that is often resistant to many cytotoxic drugs and is associated with significantly decreased disease-free survival (DFS) and OS [138, 197, 257]. In recent years a monoclonal antibody to *HER2* known as trastuzumab (Herceptin, Genentech) has become available and proven very effective in treating patients with *HER2* positive tumours. Trastuzumab binds to *HER2*, blocking the growth-stimulating intracellular signalling, decreasing cellular repair mechanisms following chemo- and radio-therapy and possibly improving apoptotic capacity [258]. However, eligibility for treatment with trastuzumab is usually based entirely on the *HER2* status of the primary tumour. Further, studies have shown that less than half of patients with an overexpression of *HER2* in the primary tumour respond to trastuzumab, whether given alone or in combination with chemotherapy [258]. Newer *HER2* blockers such as lapatinib and pertuzumab are being used in conjuction with trastuzumab and proving beneficial in clinical trials [259, 260].

As with HR status, many recent studies have demonstrated that *HER2* status differs between primary and metastatic tumours [197, 261]. Liedtke et al. [239] report a discordance of 13.6 % between primary and metastatic tissue, which is similar to the 14.4 % reported by Nishimura et al. [244]. Amir et al. [243] however, report only a 5.5 % discordance rate, though notably they demonstrate a greater loss than gain of *HER2* expression, with 12.5 % of *HER2*^+^ primary tumour patients having *HER2*^−^ metastases. Interestingly, a study by Carlsson et al. [258] examining *HER2* status in the primary tumour and lymph nodes of 47 patients with distant metastases, found no ‘drastic’ changes in *HER2* expression (i.e. change sufficient enough to alter *HER2* status classification). Unfortunately the *HER2* status of the distant metastases was not obtained as part of the investigation [258]. There is also the suggestion that *HER2* status may change during treatment. This is exemplified in a study by Apostolaki et al. [262], who assessed a cohort of 214 early stage breast cancer patients and found that 8 of 161 prechemotherapy *HER2*− patients had become *HER2*+ following treatment, which was associated with a worse DFS. The functional significance of these observations are brought home by the finding that a number of patients treated with and responding to trastuzumab were found in a subsequent centralised review to be false positive for HER2 in the primary tumour, suggesting that their disseminated disease may have gained HER2 expression [263]. Importantly, some were found to respond to the *HER2*-targeted therapy.

Issues exist here in regards to long term therapy decisions being based on primary tumour pathology, as well as issues associated with performing repeated biopsies of metastases, as described in the preceding section. Once again MRD provides a possible alternative method for patient monitoring and treatment planning. It has been seen in CTCs/DTCs, similarly to HR status, that a greater discordance is observed in the *HER2* status when compared with the primary tumour. Fehm et al. [245] report *HER2* discordance of 47 %, which is substantially lower than that seen for the HRs, but remains significantly higher than that seen between primary and metastatic tissue. The discordance of *HER2* status between CTCs and primary tumour has been demonstrated in other work, which also indicates that the phenomenon of heterogeneity between CTCs within individual patients, in regards to *HER2*, also exists as it does for *ERα*/*PgR* [264, 265].

This discordance in *HER2* expression between primary tumour and MRD has clinical significance, as a study by Apostolaki et al. [262] demonstrated that the presence of *HER2* mRNA in CTCs enriched from patient blood after they had undergone adjuvant chemotherapy was associated with decreased DFS. Multivariate statistical analysis in the study revealed that the detection of *HER2*^+^ CTCs was an independent negative prognostic factor in relation to DFS. Wulfing et al. [266] were able to demonstrate this prior to the above study, however in addition to a reduced DFS they also found an association with reduced OS. It was the work of Wasserman et al. [267] that set up the technical foundation for the development of a PCR platform for the assessment of HER2 status in MRD, on which the aforementioned studies were subsequently developed. There have been a number of publications since assessing *HER2* status in CTCs compared to the primary tumour and the persistence of these cells during treatment and its relation to poor outcome [268, 269]. *HER2*^+^ DTCs have been demonstrated to be more common in MBC patients when compared to non-metastatic patients irrespective of primary tumour HER2 status, and like their CTC counterpart these cells have been found to correlate with worse prognostic outcome [270, 271]. These publications by Pantel et al. [270] and Braun et al. [271] make the inference that the subset of DTCs that behave this way was due to; (i) the role of HER2 in modulating cell surface adhesion molecule-extracellular matrix interactions, and (ii) variable response to chemotherapeutic agents at least partly due to being in an indolent state of growth. As a consequence, the clinical implication is that this subset of cells contributes to the initial metastasis of breast cancer but also poses a risk of relapse following treatment and thus is an ideal target. The effectiveness of targeting the *HER2* antigen with trastuzumab on residual CTCs/DTCs that are resilient to standard systemic therapy post-surgery has been demonstrated [272]. Currently there is a randomised clinical trial underway in Europe (DETECT III trial), where women with *HER2* negative advanced breast cancer bearing *HER2* positive CTCs/DTCs are sorted into either a standard treatment group or a group that receives lapatinib in addition to standard care. This is now being run in conjunction with the DETECT IV trial, whereby anti-mitotic therapies are to be tested within the same cohort of recruited patients. Another European clinical trial is underway, recruiting women with *HER2* negative primary breast cancer who have persistent CTCs even after systemic therapy and surgery (TREAT-CTC trial). The patients are allocated into a standard care group, or a group that receives trastuzumab in addition to standard care. The aforementioned publications and trials are great examples of how work involved in the characterisation of CTCs/DTCs has helped identify patient populations at greater risk and also opened avenues for therapeutic intervention depending on the behaviour of these cells in a given patient.

## Survival mechanisms: platelet clumping, immunity and circulating tumour microemboli

### Platelet clumping

CTCs have been shown to form aggregates with platelets in the blood and this may confer several mechanisms of survival and increased metastatic potential [273, 274]. The effects of the interaction between CTCs and platelets are reciprocal; CTCs stimulate platelet aggregation and also excrete factors that activate platelets whilst activated platelets secrete growth factors, which in turn impact tumour growth and extravasation of CTCs or circulating tumour microemboli (CTM) [273, 274]. Platelets may provide a physical ‘cloak’ for the tumour cells, preventing access by immune cells that would destroy them. Furthermore the CTC and platelet aggregation is thought to confer protection for the CTCs against the high shear forces which occur in the blood [274]. Studies have also shown that platelets, activated by their aggregation with CTCs, secrete soluble factors including *TGFβ*, which downregulates the expression of *NKG2D* (natural killer group 2, member D), the activating immunoreceptor of natural killer (NK) cells, thereby impairing their immune function [275–277]. It has been suggested by studies in animal models that anti-platelet therapy may reduce the rate of metastases [275, 278]. The study by Yu et al. [133], as mentioned in a previous section, implicated the transforming growth factor beta (*TGFβ*) signalling pathway in their study of the metastable phenotype on CTCs, which is known to play a role in EMT. Therefore the research regarding platelet-mediated *TGFβ* stimulation of CTCs provides a candidate explanation for the occurrence of EMP in MRD, and is supported by observations from Labelle et al. [279] of platelet induced EMT being mediated by *TGFβ* and *NF*-*κB*. Additionally, the interaction between CTCs and endothelial cells, which may play a role in the extravasation of CTCs at metastatic sites, is also facilitated by platelet attachment to CTCs [280].

### Immunity

Multiple studies have assessed CTC/DTC counts several times over the course of systemic therapy, such as that by Hayes et al. [281], and it has been generally noted that patients with persistent MRD following therapy are more likely to have worse outcome [202, 282, 283]. This association has been reinforced by the single biggest study on prognostic outcome relative to CTC counts in non-metastatic breast cancer patients undergoing chemotherapy; patients who were CTC positive before therapy had worse OS, DFS, distant DFS, and breast cancer-specific survival. Meta-analyses on both CTCs [284] and DTCs [46] have demonstrated the independent negative prognostic value of CTC/DTC counts. Moreover, there has been work suggesting that in addition to the value of the presence/number of CTCs/DTCs, the dynamics of change in CTC burden over the course of therapy is also important [285]. One interpretation of this data is possibly that the detrimental effects of rigorous chemo- and radio-therapies on a patient’s immune system could contribute towards MRD survival via an immunosuppressive function. Having stated this, given that declines in CTC numbers have also been seen following therapy and are associated with better outcome, substantiating this claim becomes difficult. Further, work by Muller et al. [286] revealed that patients that went into remission following therapy were not necessarily consistently positive for CTCs, in fact most were either always negative or positive during only a single follow-up test, although the exact dynamics of when the positive result occurred and any technical limitations do need to be considered. However, in theory the interaction between platelets and MRD that potentially occurs alongside and/or drives an EMT, supplemented by systemic therapies, may contribute towards evasion of immune surveillance and promote immunosuppression. Intriguingly, it has been shown under culture conditions that cancer cells swiftly interact with platelets in a bid to transfer host MHC class I molecules onto their surface, effectively masking them and enabling evasion of NK cells, as well as the potential to disrupt T-cell mediated acquired immunity [287]. Knutson et al. [288] undertook a study that also demonstrated the persistence of cancer cells that underwent immuno-editing. They attempted to grow the mouse mammary carcinoma (MMC) cell line, originally derived from *HER2* transgenic mice, in Friend leukaemia virus 1b (FVB/N) parental mice and found after some time that a more resilient immuno-edited population arose, which was determined to have gained a mesenchymal phenotype. Considering the implication of this EMT, its association with immuno-editing, the occurrence of immuno-editing following interaction with platelets, as well as platelet-derived *TGFβ*-driven EMT; it should be noted that *SNAIL*-mediated EMT has been shown to induce immunosuppression through altered expression of immunoreactive epitopes [289]. Hence it may be that immuno-editing by cancer cells occurs alongside that which is happening with platelets, both of which cumulatively push EMP, the result being enhanced immune suppression and evasion.

### Circulating tumour microemboli

Although the extent of study on this phenomenon is extremely limited, it has been reported that CTCs can exist as clumps of contiguous cells in the circulation, which have recently been named CTM—or ‘CTC clusters’. The earliest research to demonstrate CTMs in clinical patients was performed on blood samples from colorectal cancer patients [290], which was only preceded by studies in animal models [291, 292]. Molnar et al. [290] found that CTCs can indeed persist in clusters or doublets of cells that were either entirely CK positive or a mixture of CK^+^ and CK^−^ cells. This was followed by Marrinucci et al. [169], who were investigating the morphologic heterogeneity of CTCs in a MBC patient and also noted the presence of CTC clusters. Their observations coincided with those by the Molnar group in regards to the clusters containing a mixture of cells, but moreover that the CTCs within these clusters displayed variation in their size and morphology [169]. Subsequent to this, Cho et al. [293] expanded on preceding work by investigating CTMs in a range of metastatic epithelial cancers and found at least one cluster in approximately 50 % of breast and lung cancer patients, 22 % of pancreatic cancer patients and 93 % of prostate cancer patients. These clusters are proposed to have survival and proliferative advantages over single CTC in the circulation [280]. A study by Hou et al. [294] examining CTCs, apoptotic CTCs and CTMs in small-cell lung cancer, found that apoptotic CTCs ranged from 0.2 to 20 % of overall CTC numbers but that none of the cells comprising CTMs exhibited apoptotic morphology. They also demonstrated that although the cell proliferation marker Ki67 expression was detected in a portion of solitary CTCs from patient samples, all CTMs were negative for Ki67, a finding that was also seen in a separate study on NSCLC by the same group [295]. They found that CTMs when injected into mice, demonstrated a higher metastatic potential than solitary CTCs. They suggest that the absence of apoptotic cells and of proliferating cells within CTMs give the clusters a survival advantage, protecting them from anoikis and making them relatively resistant to chemotherapy and radiotherapy [294]. Most recently, Aceto et al. [296] showed that CTMs were oligoclonal, and thus derived from the primary tumour, and that their presence in the blood of breast cancer patients correlated with poorer OS and PFS.

## Perspectives

The potential role of CTCs and DTCs (which together with therapy resilient cancer cells are collectively termed MRD) in the clinical setting is progressing towards the possibility of being used as part of routine care. CTCs in particular provide an exciting ‘liquid biopsy’ prospect as blood sample collection is both minimally invasive and very quick, providing access to disseminating cancer cells. In either instance, the idea that these cells can provide an important genomic guide to the disseminated disease, and can be monitored at regular intervals during the course of cancer treatment would allow for constant, up-to-date, optimised and personalised treatment planning and strategies. For example, the advance in short term culture of CTCs using recent methods showed that it was possible in ~80 % of MBC cases and ~40 % of EBC cases, potentially allowing some drug assessment alongside patient treatment [297]. Whilst the presence of MRD, as well as its persistence during the course of systemic therapy, has yielded associations with worse prognostic outcome, to date evidence of clinical utility is insufficient to warrant routine investigation of MRD during standard clinical care as assessed by the ASCO and SWOG S0500 study [199, 202]. The CirCe-01 clinical utility trial in France is ongoing, having also shown strong prognostic power of CTC enumeration as an initial finding [203]. What is clear is that CTCs and DTCs can be different from one another, both between patients and within the same patient, and this provides explanation as to why variable outcomes have been observed amongst patients positive for CTCs/DTCs, which was a key issue identified by the ASCO consensus panel [199]. Thus advancing our knowledge on the biology of these cells has become the central focal point and the key to their application. Whilst great technical challenges exist in this realm of research, much has still come forth about MRD in a relatively short period of time relating to aspects such as; heterogeneity, discordance with primary tumour and metastasis, EMT, CSCs, therapy resistance, survival, behaviour with neighbouring cells and the microenvironment, as well as changes in our understanding on the nature of metastasis. The advances that have been made in our understanding of CTCs and DTCs in the last two decades in unprecedented; there are more than 20,000 publications in Medline on CTCs alone. The prognostic relationship between CTC presence/enumeration by numerous methodologies, none more robust than the Veridex CellSearch system despite its reliance on epithelial perseverance, is undeniable, and new horizons in terms of diagnostics and therapeutic targeting is strongly compelling. So in order for MRD to progress into the realm of routine clinical practice, several areas must (and are currently) be addressed; (i) work characterising the molecular and phenotypic behaviour of MRD must continue in order to reliably identify subpopulations of cells that are truly responsible for disease progression in carcinoma patients that otherwise would simply be deemed “CTC/DTC positive”, (ii) evidence of benefit towards patient care with respect to therapeutic management must be demonstrated through identification of dangerous MRD subpopulations over the course of treatment—this may soon be the case upon completion of the CirCe-01, DETECT III/IV and TREAT-CTC trials, (iii) the approach used to isolate and identify CTCs/DTCs must become globally standardised—there are currently a vast range of techniques and parameters used identify CTCs/DTCs [196, 298–301], and (iv) a rapid and reliable method of expansion of MRD must be developed for the testing and identification of effective chemotherapeutic agents. Applying all these advances in knowledge and technique would allow for administration of the most useful medication while avoiding unnecessary exposure of the patient to ineffective drugs, all in a timely and universal fashion.
